# e-Health in cardiology: How to cure the infarcted innovation implementation

**DOI:** 10.1007/s12471-018-1205-2

**Published:** 2018-11-27

**Authors:** I. I. Tulevski

**Affiliations:** location Amsterdam Zuid, Cardiology Centers of the Netherlands, Amsterdam, The Netherlands

*Isn’t it strange that most of the things in life we can arrange on demand, but not the most precious one, our health*

## Why?

The Dutch healthcare system and cardiology in particular have an excellent international reputation. Innovation, research and the quality of the healthcare belong to the world’s top. However, in many ways, the implementation of necessary system improvements is slow.

It is a common opinion that scalable healthcare models are needed in order to cope with the ever increasing demand, mainly in chronic disease management. Life expectancy is increasing due to major improvements in the treatment of life-threatening conditions, the living environment is getting safer, thereby improving longevity, but also increasing healthcare need. The impact of this trend will be enormous: the system ‘as is’ cannot absorb the demand. Traditionally, hospitals are meant to deliver acute and subacute care and intermittent admissions, but lack facilities and means to provide chronic care. The waiting lists are already too long and the bottlenecks are outpatient care, postoperative/intensive care and emergency room capacity. Investing in more bricks will not alleviate the problem, as there is already a worrisome shortage of trained labour force. Even if this were an option, the effect on the healthcare budget would be enormous, probably making healthcare unaffordable.

The current system is sufficient but non sustainable and has a few serious challenges to overcome. In the Netherlands we are very proud of the solidarity principle, where good healthcare is seen as a social right, and we all want to safeguard this common good. However, a budget system is imposed as we cannot spend more than we earn, hence limiting the scale of healthcare consumption.

The patients are unaware of the costs of their treatment and have the perception of healthcare as an ultimate social right, demanding the best there is. The reimbursement system is stimulating doctors to perform medical procedures, diagnostics and hospital admissions. One could say a match made in heaven to increase the price ticket we all pay for.

In order to keep the costs under control, the government has created a vast administrative system which royally surpasses its aim. Substantial funds are spent in bureaucratisation of healthcare with no single proof of improvement of the system. To the contrary, both healthcare providers and patients have experienced this as highly cumbersome and undesirable.

Innovative concepts such as e‑Health and telemonitoring are costly, especially in the initial phase due to low volumes. Introducing telemonitoring in the current healthcare finance system has a negative effect on hospital budgets: on the one hand costly investments to acquire the system and relatively high operational costs to maintain the system, and on the other hand it leads to less declarable activities as the patient will eventually stay away from the hospital. This is a very important hurdle to scaling up telemonitoring systems in the Netherlands.

Last but not least, a hurdle to overcome is the financial partition between primary and secondary healthcare services. Insurance companies are having difficulty in effectively relocating the budgets between the healthcare institutions, thereby unintentionally limiting the innovators and rewarding the status quo.

It is very unlikely that the current reimbursement system will change in the near future, as transformation is very costly and there is no proof yet that another system could be more efficient and sustainable, while maintaining the solidarity principle. In order to take the necessary steps to transform the current health system into a modern and sustainable system, where healthcare supply will be aligned with the demand and the investments made in clicks and not in bricks, we all need to start with a change in mindset.

## How?

The human body is a continuous source of biodata, and currently we obtain the data in healthcare silos, meaning that the patient has to be physically present. This strategy has a lot of shortcomings; it is costly both for the healthcare system, and for the patient, resulting in work absence, travelling costs, and parking costs, is not scalable as the care is being delivered ‘by two hands on one patient’, and is only possible by a direct interaction between the patient and the doctor at the same location. This can all be elegantly organised if a patient uses a wearable connected to a specific application delivering the data to a command centre. Interaction between the streaming data (sent by the patient) and the electronic medical record is essential for the scalability of the program. This interaction can distillate relevant data and generate personalized alarms. When needed, a telephone or video connection can be established between the patient and dedicated medical staff. This strategy makes healthcare scalable, affordable, feasible, and independent of the location and time, thereby making the patient more mobile and independent, and giving better continuous or on-demand data to the cardiologist. The open communication channel between patients and cardiologists offers a unique window of opportunity for primary and secondary prevention, education, improved compliance and much better assistance to the general practitioner to manage more patients with the right support.

## What?

In general, most patients with a chronic cardiac condition are eligible to be included in some sort of telemonitoring program. In the initial phases, patients with therapy-resistant hypertension, rhythm disturbances, heart failure, congenital heart disease and preoperative and postoperative follow-up will benefit the most. There are lots of opportunities, domestic and international. Healthcare can be seen as a commodity with a worldwide demand, whose quality and price are probably the two most important reasons why future patients and payers will select their health providers. Modern healthcare will be partially independent of the geographic regions and can be exported, thereby generating revenues at a distance. Without doubt, medical technology is indispensable to people’s health and improved quality of life. Technology already has an enormous impact on healthcare. From improved operational efficiency to standards in patient care, healthcare transformation has enhanced the entire experience for both patients and medical professionals. But this is just the dawn: the big data, Nano technology and genomics will irreversibly and to an unimaginably large extent change the medical world and its services. The medicine of the future will be highly accessible, high tech at low costs, predictable and preventable. Dutch cardiology can play an important role on the world stage, but only if we dare to step out of the comfort zone and implement innovation in practice.

In this special issue of the Netherlands Heart Journal, the reader can appreciate the efforts of pioneering Dutch cardiologists showing examples of how to make healthcare sustainable and independent of location and time, reaching for the ultimate goal of healthcare on demand, feasible, affordable and scalable. I sincerely hope that you will enjoy reading this special edition and join these authors in their novel strive for a change in mindset and fast implementation of the proven innovative concepts we come across on a regular basis.

